# Visceral Leishmaniasis and Arsenic: An Ancient Poison Contributing to Antimonial Treatment Failure in the Indian Subcontinent?

**DOI:** 10.1371/journal.pntd.0001227

**Published:** 2011-09-27

**Authors:** Meghan R. Perry, Susan Wyllie, Vijay Kumar Prajapati, Joerg Feldmann, Shyam Sundar, Marleen Boelaert, Alan H. Fairlamb

**Affiliations:** 1 Division of Biological Chemistry and Drug Discovery, College of Life Sciences, University of Dundee, Dundee, Scotland, United Kingdom; 2 Infectious Disease Research Laboratory, Department of Medicine, Institute of Medical Sciences, Banaras Hindu University, Varanasi, India; 3 College of Physical Sciences–Chemistry, Trace Element Speciation Laboratory, Meston Walk, University of Aberdeen, Aberdeen, Scotland, United Kingdom; 4 Epidemiology & Disease Control Unit, Department of Public Health, Prince Leopold Institute of Tropical Medicine, Antwerp, Belgium; Institute of Tropical Medicine, Belgium

## Introduction

**Figure 1:**
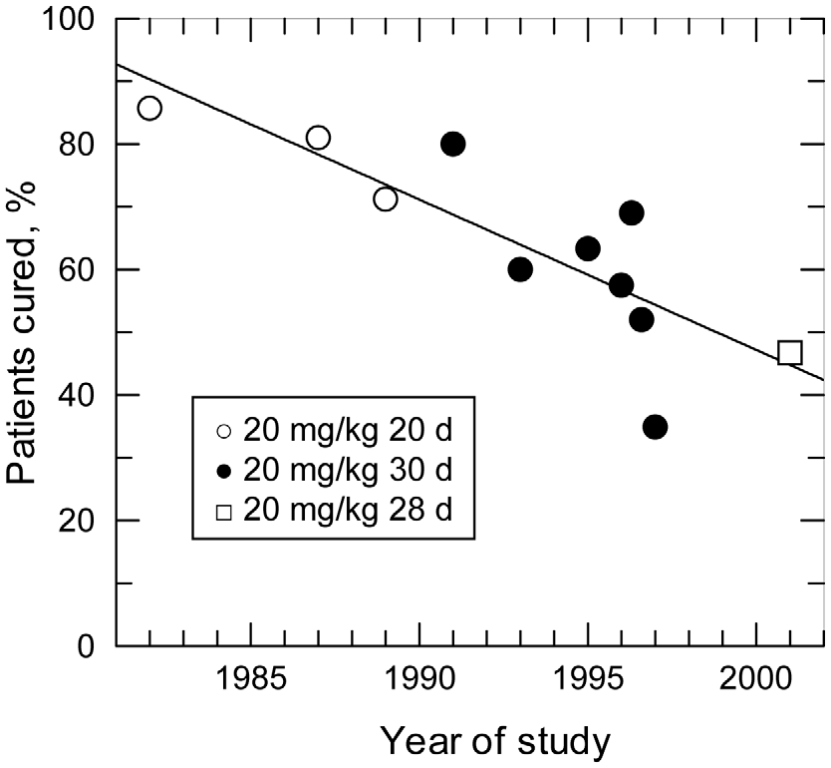
Results of consecutive clinical studies of antimonials at 20 mg/kg dosing in Bihar, India. Graph drawn from data presented in tabular form in a systematic review of clinical trials done in India between 1980 and 2004 [2]. Doses received are indicated in the figure key. Linear regression correlation coefficient  =  −0.831.

Antimony and arsenic are elements that have a long history of use as poisons, therapeutic agents, or cosmetics. For over a century, compounds containing pentavalent antimony (antimonials) have formed the basis of treatment of the leishmaniases worldwide. Antimonial preparations remain first-line drugs for visceral leishmaniasis in Sub-Saharan Africa and Brazil [1], but in the hyperendemic state of Bihar, India, the cure rate of antimonial compounds has declined over the past 30 years from over 85% to less than 50% (Figure 1) [2] and resistance in parasites has been demonstrated [3]. This marked decrease in efficacy has been attributed to long-term, widespread misuse of antimonials, with patients undergoing inappropriate treatment courses often administered by the largely unregulated Indian private health care system [4], [5]. Here we propose an additional hypothesis to explain the substantially lower efficacy of antimony in Bihar compared to other regions in the world.

Since the 1970s, millions of inhabitants of Asia have been chronically exposed to naturally occurring arsenic on a daily basis through the large-scale insertion of multiple shallow tubewells which were originally installed for provision of clean and safe drinking water [6]. Antimony and arsenic are metalloids belonging to Group 15 of the periodic table that share many structural and chemical properties [7]. Antimony resistance in *Leishmania* parasites can be induced experimentally by exposure to stepwise increasing concentrations of sublethal concentrations of trivalent arsenite in culture [8]. If an individual who is chronically exposed to arsenic is infected with *Leishmania*, the parasites would be exposed to arsenic due to its presence within organs of the lymphoreticular system [9]. This could lead to the development of an arsenic-resistant *Leishmania* strain that would be cross-resistant to antimonial therapy. This viewpoint article will expand on, and discuss the evidence for, the possible contribution of arsenic to decreased antimonial efficacy in Bihar.

## Visceral Leishmaniasis

The Leishmaniases are a group of diseases caused by infection with the protozoan parasite *Leishmania*, transmitted by the sandfly. The most serious form of the disease is visceral leishmaniasis, which results from infection with *Leishmania donovani* and *Leishmania infantum.* The amastigote form replicates in macrophages of the liver, spleen, and bone marrow, causing persistent fever, hepatosplenomegaly, weight loss, and pancytopenia. If untreated it is eventually fatal [10] and is thought to account for 41,000 deaths per year [1].

Approximately 60% of the worldwide incidence of visceral leishmaniasis is in the Indian subcontinent [11], where it has an anthroponotic transmission which follows a cluster pattern. Ninety percent of India's disease burden is thought to occur in Bihar [12], and in 2009, the incidence was estimated at 22 per 10,000 of the population [13]. Supported by the World Health Organisation, the governments of India, Nepal, and Bangladesh are currently implementing an elimination programme that aims to reduce the incidence of the disease to less than 1 per 10,000 by 2015 [13].

## Resistance and Epidemiology

Visceral leishmaniasis was almost eliminated in India in the 1950s and 1960s as a secondary consequence of the National Malaria Eradication programme. After the programme's termination the sandfly vector population increased, resulting in a resurgence of visceral leishmaniasis reaching epidemic proportions in the late 1970s [14]. Primary unresponsiveness to antimonials became evident during this epidemic when physicians found the prior standard 6-day antimonial regime to be “grossly inadequate” [15]. As a result, a 20-day regime at 10 mg/kg was introduced which was further increased in subsequent years up to 20 mg/kg for 30 days to try to reduce the increasingly high rate of relapse [16]. Despite this, cure rates with antimonial treatment continued to fall [2]. Subsequently, new, effective therapies, such as oral miltefosine and intravenous amphotericin B deoxycholate, have been incorporated into routine clinical practice and now form the treatment backbone of the visceral leishmaniasis elimination programme [11].

The emergence of decreased antimonial efficacy within the epidemic occurred in the years directly following the large-scale insertion of shallow tubewells across Asia in the early 1970s. The natural occurrence of high levels of arsenic in the groundwater accessed by many of these tubewells was only discovered in Bihar in 2002 [17], although it is believed to have been present since the tubewells were inserted [6]. This late finding came despite Bihar's close geographic proximity to West Bengal and Bangladesh, where the occurrence of mass arsenic poisoning was identified in the 1980s and 1990s, respectively [6]. By 2007 UNICEF had tested 66,623 tubewells in Bihar and found 28.9% had arsenic levels greater than the WHO standard of 10 µg/L, affecting at least 7.2 million of the population [6].

**Figure 2:**
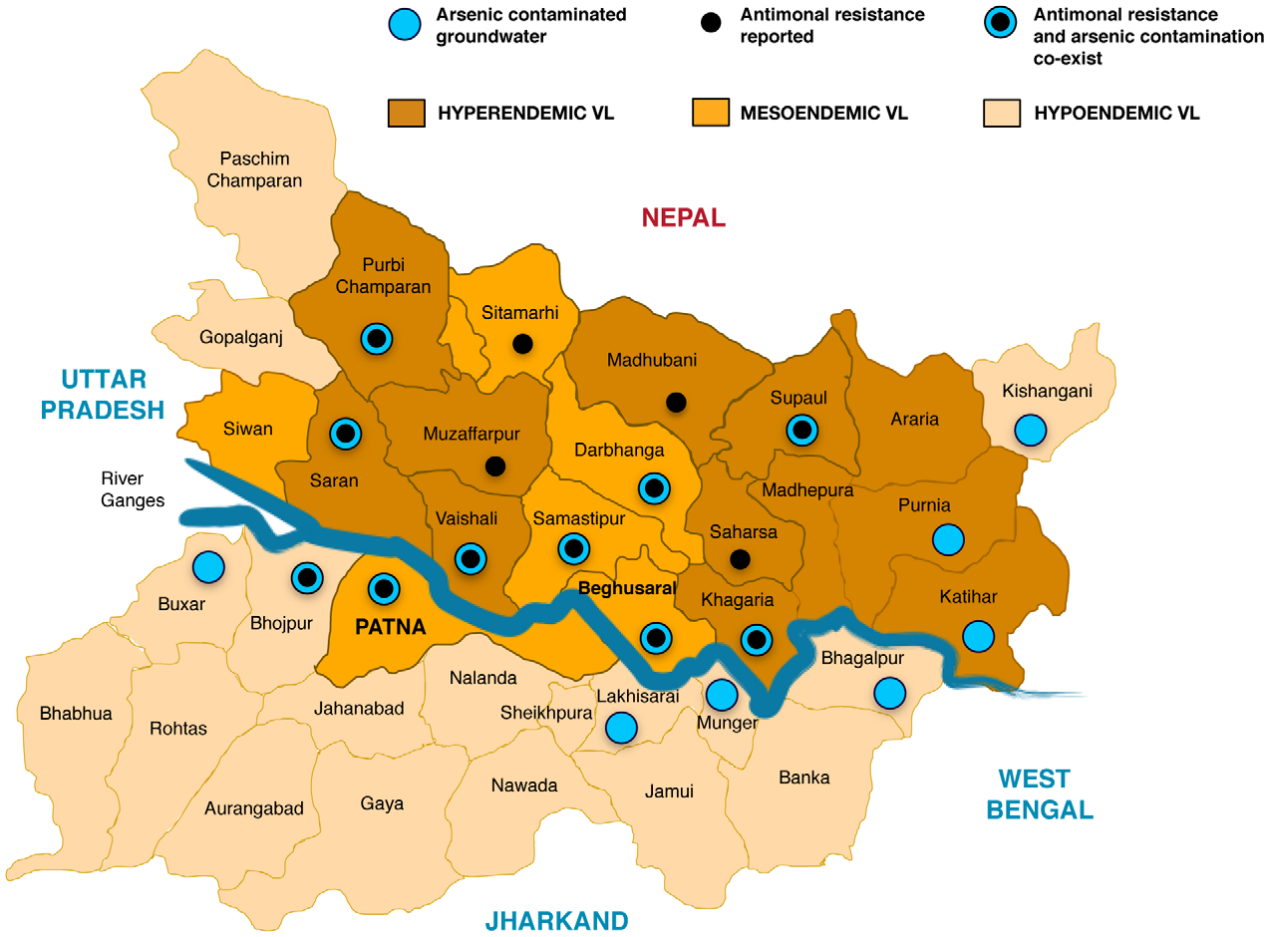
Map of Bihar showing district distribution of visceral leishmaniasis endemicity, antimonial resistance, and arsenic contamination of the groundwater. The map is based on published data [4], [6], [44]–[46] with additional data from http://www.indiawaterportal.org/node/10925 and http://www.soesju.org/arsenic/groundwater_bihar.htm, both last accessed 12/20/2010. Data for Muzaffarpur are from personal correspondence with Dr. Ashok Ghosh, A.N. College, Patna.

The Indian subcontinent is the only place worldwide where endemic visceral leishmaniasis and significant arsenic contamination of the groundwater co-exist [6], and it is from Bihar and Nepal that the highest rates of antimonial treatment failures are found [4], [18]. In the map of Bihar in Figure 2, the available information from journals, websites, and published databases on the incidence of visceral leishmaniasis, antimonial resistance, and arsenic contamination of the groundwater has been collated. This epidemiological data must be interpreted in the context of an estimated 73%–90% under-reporting of visceral leishmaniasis [19] and the incomplete analysis of tubewell water throughout Bihar. However, it does demonstrate 10 endemic VL districts where part of the population's consumption of water from arsenic contaminated tubewells could have resulted in the induction of antimonial resistance of *Leishmania* parasites through arsenic exposure via their human host. Our hypothesis has parallels with the subtherapeutic exposure of South American, South East Asian, and African populations to chloroquine through medicated salt in the 1950s and 1960s, which is thought to have contributed to the spread of chloroquine resistant malaria [20].

In the northern areas of Bihar there is less reported arsenic contamination of groundwater [6], but antimonial resistance is still present at high levels. Worker migration between districts is common in Bihar [21] and dissemination of resistant strains from areas of high level arsenic exposure along the Ganges to the north through clusters of dense visceral leishmaniasis incidence would account for this epidemiological pattern. This is supported by known high rates of transmission [19], demonstrable superior fitness of clinical resistant strains [22], and long-term stability of resistant laboratory strains passaged in the absence of metalloid pressure [23], [24].

In neighbouring Bangladesh, where minimal antimonial resistance has been reported, high-level arsenic contamination is mainly in the south of the country [6], whereas visceral leishmaniasis is mainly found in the north [25]. In other endemic countries such as Sudan and Brazil, where there is no reported arsenic contamination of the groundwater, efficacy rates are greater than 95% [1]. In Sudan and Brazil prevalent issues such as intermittent poor drug availability, low treatment compliance, and civil unrest could also have led to inadequate or interrupted treatment courses. The current thinking that drug misuse is solely responsible for the dramatic development of resistance in Bihar does not account for the presence of these same issues in countries where antimonials remain an effective treatment.

## Resistance and Biochemistry

Chronic arsenic exposure through drinking water is associated with an increased mortality rate [26], particularly from cancer, cardiovascular diseases, and infections [27]. Arsenic exists in groundwater as arsenate (As^V^) or arsenite (As^III^), which, following ingestion, is metabolised in the liver via a series of reduction and subsequent oxidative methylation steps to methylarsonic acid and dimethylarsinic acid. The majority is excreted in those forms over subsequent days in the urine [28], but a small proportion accumulates in the skin, hair, and nails [28] mainly as As^III^ and As^V^ [29] and, significantly for this hypothesis, in the liver [9], [30]. There are no data currently available in chronically arsenic exposed humans on arsenic levels in the spleen or bone marrow, the other organs where parasites reside in visceral leishmaniasis.

In the livers of laboratory animals, total arsenic levels (both methylated and inorganic forms) have been shown to be related to dose and duration of exposure. BALB/c mice orally dosed with 150 µg of arsenite for 6 out of 7 days a week for a year show an almost 10-fold increase in total hepatic arsenic from <0.5 mg/kg to ∼4.5 mg/kg [30]. Comparable total hepatic arsenic contents, ranging from undetectable levels up to 6 mg/kg, were found in liver biopsies performed on a small cohort of 13 hepatomegalic patients from West Bengal, who had been environmentally exposed to arsenic levels of 220 to 2,000 µg/L for 1 to 20 years [9].

Should arsenite levels in spleen, bone marrow, and liver macrophages be freely bioavailable and similar to total hepatic arsenic content, then it is theoretically possible that arsenite could kill *Leishmania* parasites and thus have a protective effect against infection. More likely, however, is that arsenic levels within macrophages of chronically arsenic exposed patients are sublethal, thus creating an environment where resistance can develop. Developing arsenical resistance through selective environmental exposure is a well-researched and established mechanism in microorganisms [31]. Since the incubation period of visceral leishmaniasis ranges from weeks to months [10], parasites could develop tolerance to arsenite by evolving resistance mechanisms that would also be effective against antimonial compounds when administered as treatment. Although there are no direct reports of uptake of arsenic into macrophages, it is plausible to assume that this does occur because pentavalent antimony is known to be taken up by macrophages [32] and both trivalent arsenic and antimony have be shown to be taken up, via aquaglyceroporin 9, by undifferentiated human leukaemia cells [33].

There has been extensive research on the mechanisms by which *Leishmania* parasites acquire resistance to antimonial preparations [4]. This research initially involved generation of antimonial-resistant strains through stepwise exposure of *Leishmania* parasites to arsenite in vitro in order to study an arsenite-thiol conjugate transporter PgpA [34], which was subsequently found to be an important vacuolar sequestration mechanism involved in *Leishmania* antimonial resistance [35]. Both arsenite and antimony have since been used to select resistance and many common mechanisms have been demonstrated [4] that have subsequently been identified in antimonial-resistant clinical isolates [36], [37].

The full mechanism of action of antimony itself is incompletely understood. It is known to be active in its trivalent form [38], but is administered as a prodrug in pentavalent form and reduced either in the macrophage, in the amastigote, or both [4]. In a sensitive parasite lineage trivalent forms of arsenic and antimony accumulate within the parasite, but this accumulation is found to be reduced in resistant strains [39]. Both antimony and arsenic in their molecular form as antimonite (Sb(OH)_3_) and arsenite (As(OH)_3_) react with sulphydryl groups and are thought to be leishmanicidal through depletion and interference with the parasite's unique thiol system [38], [40], which is dependent on the dithiol trypanothione. This system is responsible for maintaining the parasite's redox balance and thus its ability to defend against oxidative stress. In both laboratory and clinical-resistant parasite isolates an increase in protective thiols [39] and overexpression of associated enzymes has been reported [41].

## Further Investigation

Establishing whether arsenic exposure could have contributed to antimonial resistance is a process that will require the integration of epidemiological, clinical, biological, and analytical chemistry data. Due to high levels of treatment failure antimonial compounds are no longer the first-line treatment in Bihar. Thus, assessment of resistance relies on retrospective clinical data or upon the analysis of clinical parasite isolates using in vitro assays. The latter can be an unreliable indication of clinical response [42] and produce variable results depending on the methods used [43]. Furthermore, if resistance is identified in a *Leishmania* clinical isolate it is not currently possible to determine whether this is due to prior arsenic or antimonial exposure via the patient or whether this was transmitted from a prior human host. We hope to be able to demonstrate proof of concept in the laboratory using in vitro systems and through exposing *L. donovani* infected laboratory animals to environmentally relevant levels of arsenic in their drinking water. This laboratory work will help to guide future clinical and epidemiological research.

## Conclusion

Biochemical, historical, and geographical data indicate a significant link between arsenic contamination of the groundwater and the development of high levels of resistance to antimonial compounds found in the treatment of visceral leishmaniasis in Bihar. As antimonials are still being used widely elsewhere in the world, continued efforts into gaining full understanding of the reduced efficacy of this drug in Bihar will be beneficial in guiding future drug combination policy and treatment options.
